# Measuring newborn foot length to estimate gestational age in a high risk Northwest Ethiopian population

**DOI:** 10.1371/journal.pone.0238169

**Published:** 2020-08-27

**Authors:** Nega Dagnew, Ashenafi Tazebew, Abebe Ayinalem, Abebe Muche

**Affiliations:** 1 Department of Human Anatomy, College of Medicine and Health Sciences, University of Debretabor, Debretabor, Amhara, Ethiopia; 2 Department of Human Anatomy, College of Medicine and Health Sciences, University of Gondar, Gondar, Amhara, Ethiopia; 3 Departments of Pediatrics and Child Health, College of Medicine and Health Sciences, University of Gondar, Gondar, Amhara, Ethiopia; University of Oklahoma Health Sciences Center, UNITED STATES

## Abstract

**Introduction:**

Preterm birth is defined as all births before 37 completed weeks of gestation. Globally, the prevalence rate of preterm birth ranges from 47.5 to 137 per 1000 live births. In Ethiopia, the prevalence of preterm birth is 10.1%. Several anthropometric parameters, particularly, head circumference and foot length(FL) have been used as a proxy measure for gestational age(GA).

**Objective:**

To assess the use of newborn foot length as a screening tool to identify preterm newborns and correlation factors at the University of Gondar Comprehensive Specialized Hospital (UOG CSH), Northwest Ethiopia.

**Methods:**

Institutional based cross-sectional study design was conducted on 205 newborns admitted to a neonatal intensive care unit, UOG CSH. Systematic sampling technique was employed. Optimal cutoff newborn foot length and area under the curve (AUC) was calculated by the receiver operating characteristic curve analysis to assess the power of foot length measurement to diagnosis prematurity.

**Results:**

The mean foot length was 7.41±0.67 cm with a range of 5.4–8.6 cm. Gestational age had a significant strong positive correlation with foot length(r = 0.865). The regression equation derived was GA = 4.5*FL + 3.61. Foot length had strong power (AUC = 0.99) to differentiate preterm from term newborns. A threshold newborn foot length of ≤7.35 cm had a sensitivity and specificity of 98.5% and 96.3%, respectively to predict prematurity.

**Conclusion:**

Foot length had a high sensitivity and specificity in identifying preterm newborns, making it a reliable tool to identify preterm birth in a rural setting.

## Introduction

According to the World Health Organization (WHO), preterm birth is defined as all births before 37 completed weeks of gestation [1]. The preterm newborns can be divided as moderately preterm (33 to 36 completed weeks of gestation), very preterm (28–32 weeks) and extremely preterm (<28 weeks) [2].

Globally, 13 million babies are born preterm annually with the prevalence rate ranging from 47.5 to 137 per 1000 live births [3, 4]. According to the 2010 global report on preterm birth and stillbirth, the highest rate of preterm birth was reported in low and middle-income countries with progressively increased incidence in some middle and high-income countries [3]. Of note, the highest prevalence of preterm birth was recorded in Africa (11.9%) and North America (10.6%) [5]. The 2010 global action report on preterm birth indicated that the prevalence of preterm birth in Ethiopia was 10.1% [6]. However, there was a wide variation in the prevalence of preterm birth among the different cities in Ethiopia, with Addis Ababa (7.1%), Debre Marqos Hospital (11.6%), and Gondar referral hospital (14.3%) [7–9].

The study published in the Lancet focusing on When? Where? Why? of neonatal deaths, indicated that preterm birth accounts for 28% of direct cause of total neonatal deaths worldwide [10]. More than four-fifths of deaths due to prematurity (83.2%) occurred in the first week of life; the first day (day 0) contributed around 40%. About 8 to 10% of deaths secondary to prematurity occurred in week 2 and weeks 3 to 4 of life. Of the estimated 6.3 million children under 5 who died in 2013, 15.4% (0.965 million) were due to complications of preterm birth [11]. The etiology of preterm birth is multifactorial and involves a complex interaction between fetal, placental, uterine, and maternal factors [12]. Preterm neonates are at an increased risk for a wide range of short and long term respiratory, infectious, metabolic and neurological morbidities, with higher risks of adverse outcomes seen at lower gestational ages [13].

Gestational age is a major determinant of newborn prognosis. It can be estimated by Naegele’s formula or ultrasonography. GA estimates based on Naegele’s formula in settings with low literacy tend to have lower accuracy [14]. New Ballard Score (NBS) is a valid and reliable clinical tool for GA assessment. However, its accuracy depends on the skill of the examiner and the neonate’s condition [15]. In order to reduce global mortality of preterm birth, early identification of gestational age within 48 hours of birth, especially in differentiating preterm from full-term newborns born at home or in remote areas, is a major priority for researchers and public health practitioners. Mortality and morbidity can be prevented if preterm newborns are identified earlier and treated with simple interventions such as skin-to-skin contact or kangaroo mother care (KMC), early breastfeeding, as well as early infection prevention and treatment [16]. However, identification of preterm newborns is difficult in community settings.

As a result, an inexpensive and simple method is required to identify at-risk preterm newborns soon after birth. Foot length (FL) has been determined in neonates using simple measuring instruments and can be used as an anthropometric surrogate for estimation of GA. Neonatal anthropometric measurements are of both epidemiological and clinical use. Clinically, the anthropometric measurements are valuable tools used to detect neonates that are at higher risk of neonatal and postnatal morbidity and need for growth improvement [17, 18].

Rural areas with low literacy levels, application of Naegele’s rule and non-availability of antenatal ultrasonography and trained personnel are the limiting factors. As a result, a simple and inexpensive measurement like newborn foot length can be used to screen prematurity that has great potential for newborn survival. Measurement of newborn foot length for childbirths in resource-poor settings, especially at the primary health care unit (PHCU) has the potential to be used by birth attendants, health extension workers or parents as a screening tool to identify premature newborns in order that they can receive targeted interventions for improved survival.

To our knowledge, there is no such study assessing the use of newborn foot length measurement as a screening tool of prematurity in Ethiopia. Therefore, the objective of this study was to assess the use of newborn foot length as a screening tool to estimate gestational age and to identify preterm newborns at risk of early and late post-natal complications.

## Methods and materials

### Study period and study area

This was an institutional based cross-sectional study conducted between January 2019 and March 2019 in UOG CSH, Department of Neonatal Intensive Care Unit (NICU). The hospital has 611 beds and acts as the referral center for 12 district hospitals in the area. The NICU was also fairly equipped with an average of 10 term births/day and 2–5 preterm births/day admissions and provides a tertiary level of care. On average, there were 25–30 deliveries per day in the maternity ward from which 85–90% were full term.

In the present study, all neonates for whom consent was given by mothers and gestational age calculated by NBS between 28–42 weeks were included. Newborns with intrauterine growth restriction (IUGR) and extremely preterm (gestational age < 28 weeks old) and neonates with congenital anomalies of the foot like club foot that hinder anthropometric measurement were excluded from the study.

### Sample size determination

The sample size was obtained based on the diagnostic test formula [19], that is, the number of newborns necessary to estimate the area under the ROC curve or power of an anthropometric measurement foot length as a screening tool for identifying preterm newborns. By taking confidence interval (CI) 95%, AUC (area under the curve) = 0.95 [20], the ratio of prevalence of term to preterm subjects was 2, and margin of error was 5%. By assuming 10% non-response rate the final sample size was 205 where 68 were preterm and 137 were term newborns.

### Sampling technique

The systematic random sampling technique was employed. The average daily preterm admissions were 3. By dividing the total of 2-month admissions (180) by the final sample size (68), every second preterm newborn was measured. The first preterm newborn was selected through drawing a number from 1 up to 2 and by the lottery method, the number two was selected and continuing with every second number until the final sample size was reached. The average term daily admissions were 10. By dividing the total of 2-month admission (600) by the final sample size (136), every fourth term newborn was measured. The first term newborn was selected through drawing a number from 1 up to 4 and by lottery method, the number one was selected and continuing with every fourth number until the final sample size was reached. If a newborn does not satisfy the inclusion criteria, then the next participant was selected.

### Data collection procedures and tools

A properly designed checklist was used to collect relevant information. Medical records were reviewed for early ultrasound findings, estimated date of delivery and age. Following confirmation of the normal appearance of foot by physical examination, the study subjects were recruited to the study. Newborns were placed in the supine position and the right foot was placed in a lateral position while the ankle was held and a finger placed on the foot dorsum so as not to elicit a grasp reflex, which may shorten the measurement. The right foot was measured from the posterior aspect of the heel to the tip of the Hallux (big) or longest toe ensuring that no pressure was exerted on the soft tissue, using a plastic Vernier’s sliding caliper, calibrated to 0.1 cm precision. A balance beam neonate scale was used to measure the weight of the neonate. All infection prevention, precaution standards were used during the time of measurement. Standard precautions were also applied for measuring equipment. All the measurements were taken within 24 hours of birth. The reference standard was GA determined by NBS. The NBS was determined by assigned senior pediatric residents. FL and weight measurements were performed by 3 trained neonatal nurses. There was minimal interobserver variability. To maintain reproducibility, each measurement was repeated 2 times and the average was recorded. In addition, baseline data including GA, gender, and weight were recorded for all participants.

### Data processing and analysis

The collected data were checked for completeness, accuracy, and clarity before analysis. The data were entered into EPI Info version 7.7.1 and exported to statistical package for social sciences (SPSS) version 20 for analysis. Data were cleaned and edited before analysis. The means (± standard deviation), ranges, maximum, minimum, and the 95% confidence intervals for the mean (in order to include the true population mean in 95% of the cases) was calculated. A p-value of less than 0.05 was considered as statistically significant. Non-parametric receiver operating characteristic (ROC) curves and its coordinates using SPSS was presented for the use of foot length measured within 24 hours of birth as a screening tool of prematurity. The area under the curve (AUC) was calculated to assess the accuracy of foot length measurement for the diagnosis of prematurity. The optimal cutoff newborn foot length to predict prematurity, which is defined as the point with the highest sensitivity and specificity such that the sensitivity was at least 0.8 was analyzed by ROC curve. In other words, the optimal threshold was chosen to minimize the distance from the ROC curve to the point (0, 1), subject to the constraint that the sensitivity must be at least 0.8. The sensitivity and specificity at the chosen cut-point were computed along with 95% confidence intervals (CIs). Youden’s index (Y.I) was also calculated using a formula Y.I = (SN + SP) -1 to know which cutoff point gives maximum sensitivity and specificity for this diagnostic test. Before computing parametric tests, normality of data were checked by Shapiro-Wilk test. T-test and one-way ANOVA analysis were done to assess the significant difference in mean foot length among gender, maturity status and birth weight groups. Simple linear regression analysis was done for estimating Gestational age (GA). Pearson correlation coefficient (r) was used to identify the correlation between foot length with Gestational age, birth weight, and maternal age.

### Ethical considerations

Ethical clearance was obtained from the ethical review committee of the School of Medicine, College of Medicine and Health Sciences, University of Gondar(IRB number SOM/1076/2019). An official letter was submitted to the University of Gondar Comprehensive Specialized Hospital and Department of Pediatrics. Informed verbal consent(since most of the mothers are unable to read and write) which was approved by the ethical review committee was taken from the newborn’s mother after the objective and procedure of the study was well described and their willingness to participate in the study or not was asked to the mothers of all newborns. In addition, all information obtained from them was secured and kept confidential. All data involving measurement were collected without any risk or harm to the newborns.

## Results

### Descriptive characteristics of the study population

Two hundred four (204) newborns were enrolled in this study, of which 96(47.1%) of them were males and 108 (52.9%) were females. The gestational age of newborns was in the range of 28–42 weeks with a mean of 37.0±3.4 weeks (95% CI 36.48–37.41). The birth weight of the study subjects ranged from 1050 gm to 4900 gm, with a mean birth weight of 2627 ± 770 gm. One hundred thirty-six (66.7%) of newborns were term (Table 1).

**Table 1:** Demographic characteristics of newborn study subjects at the University of Gondar Comprehensive Specialized Hospital, 2019.

Variables		Number of cases	Weight in gm			GA (NBS)		
			Mean	Range		Mean(SD)	Rang	
				Min	Max		Min	Max
Sex	Male	96	2650.0(755.0)	1100	3820	37.1(3.3)	28	42
	Female	108	2607.3(786.5)	1050	4900	36.9(3.5)	28	42
	Total	204	2627.4(770.2)	1050	4900	37.0(3.4)	28	42
Maturity status	Preterm	68	1750.5(487.3)	1050	3800	32.9(2.5)	28	36
	Term	136	3066.0(440.0)	2000	4900	39.0(1.5)	37	42
	Total	204	2627.4(770.2)	1050	4900	37.0(3.4)	28	42

A total of 52 neonates (25.5%) were LBW whereas, 22 (10.8%) were VLBW. The mean foot length of VLBW, LBW and normal neonates were 6.40, 6.93, and 7.78 cm, respectively. VLBW neonates had a lower mean foot length than other weight groups (Table 2).

**Table 2:** Gender, weight category and maturity status based distribution with Independent sample t-test for mean difference of foot length of newborn subjects, University of Gondar Comprehensive Specialized Hospital, 2019.

Variables		Number of cases	Foot length (cm)				
			Mean (SD)	Range		Independent sample t-test	p-value
				Min	Max		
Sex	Male	96	7.38(0.65)	5.4	8.6	-0.55	0.58
	Female	108	7.43(0.69)	5.4	8.5		
	Total	204	7.41(0.68)	5.4	8.6		
Maturity status	Preterm	68	6.69(0.36)	5.4	7.7	-18.3	<0.0005
	Term	136	7.78(0.46)	6.9	8.6		
**Wt in gm.**	VLBW	22	6.40(0.36)	5.4	6.9	133.0	<0.0005
	LBW	52	6.93(0.39)	5.8	8.2		
	Normal	130	7.78(0.47)	6.9	8.6		

### Foot length measurement

Of the 204 neonates studied, the mean foot length was 7.41±0.68 cm (95% CI 7.32–7.50 cm) with a range of 5.4–8.6 cm. The preterm neonates had a mean foot length of 6.69±0.36 cm (95% CI 6.60–6.78). The range of foot length measurement in preterm newborns was 5.4–7.7cm. Of the total study subjects, 35.3% had a foot length of less than or equal to 7.3 cm (Table 2).

Two-tailed independent sample t-test analysis indicated that there was no statistically significant difference (p = 0.58) in mean foot length between male and female newborns. However, there was a statistically significant difference (p<0.0005) in mean foot length between preterm and term newborns. The mean foot length of terms was higher than preterm newborns (Table 2).

There was a statistically significant difference in mean foot length measurement between weight groups in newborn study subjects as determined by one-way ANOVA (F (2,201) = 133.01, p<0.0005). Post- hoc test (Bonferroni) revealed that there was a statistically significant difference in mean foot length between VLBW and LBW, VLBW and Normal, and LBW and normal groups. The mean foot length of normal weight newborns was higher than both LBW and VLBW groups. Similarly, the mean foot length of LBW newborns was higher than VLBW groups.

A statistically significant strong positive correlation was observed between gestational age and foot length (r = 0.865 and p<0.0005). Besides, there was a statistically significant strong positive correlation between the weight of the study subjects and foot length(r = 0.803 and p<0.0005). Also, strong positive statistically significant correlation was found between the gestational age and weight of newborn study subjects (r = 0.832 and p<0.0005). However, maternal age had no statistically significant correlation with foot length, gestational age and weight of newborns (p>0.05).

Using linear regression analysis Gestational age (GA) in weeks can be estimated using the formula:

GA=4.5*FL+3.61

**Fig 1:**
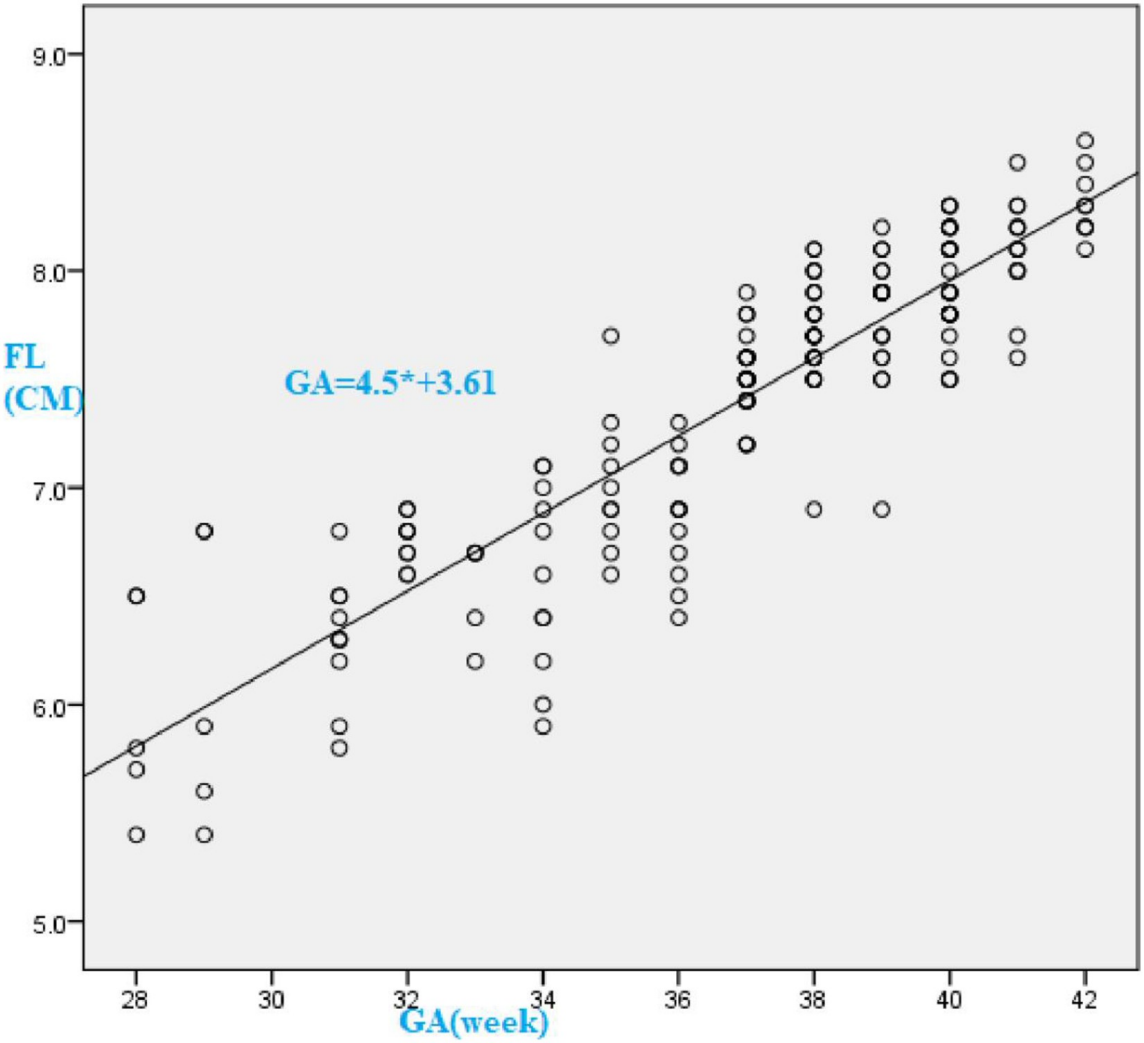
Scatterplot with regression line showing liner association of gestational age(GA) versus foot length (FL) of newborn subjects, University of Gondar Comprehensive Specialized, 2019.

There was an increase of 4.5 gestational weeks for an increase in 1 cm of foot length. There is a linear association between FL and GA (Fig 1).

### Receiver operating characteristic (ROC) curve analysis for cutoff point determination

**Fig 2:**
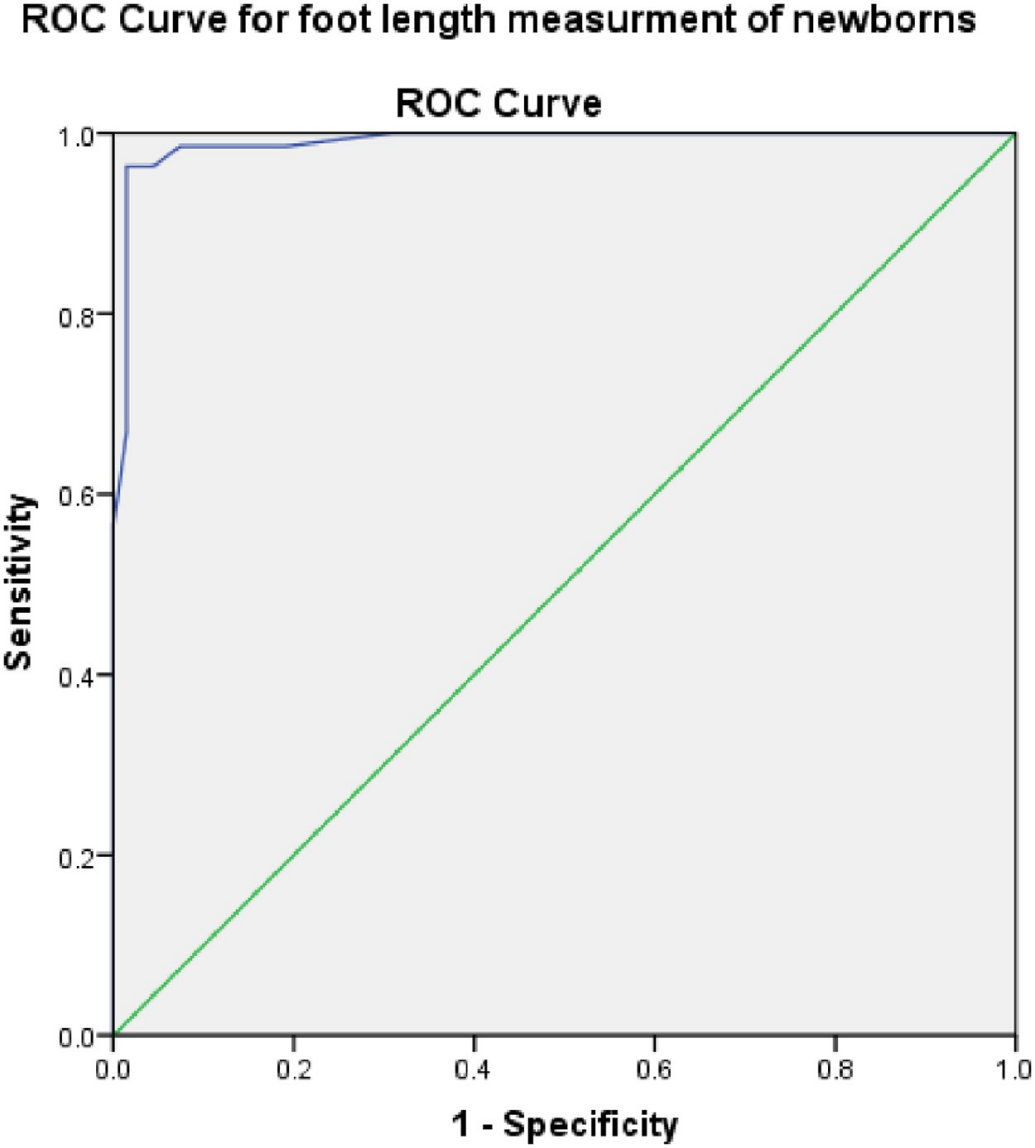
The ROC curve of foot length for predicting prematurity in 204 newborn subjects, University of Gondar Comprehensive Specialized, 2019.

The corresponding ROC curve for FL as a surrogate for prematurity less than 37 weeks was shown in Fig 2. ROC analysis to test the accuracy of foot length measurement to predict pretermity showed that it had a high area under the curve (AUC) 0.990 (95% CI 0.978–1.00). Foot length had a strong classification power to differentiate preterms from terms. It was highly accurate and had a statistically significant power to differentiate preterms from term newborns (p<0.0005). The possible operational cutoff points was determined by calculating a Youden’s index. The optimal cutoff point was 7.35 cm with sensitivity and specificity of 98.5% and 96.3%, respectively.

The point with the highest Youden index was selected to represent the optimal cutoff value with the highest overall accuracy for predicting prematurity. A result of Youden index revealed that the cutoff point of foot length of identifying preterm from term was 7.35 cm. In order to differentiate premature babies, newborn foot length of ≤7.35 cm had a sensitivity and specificity of 98.5% (95%CI 92.1%-99.7%) and 96.3% (95%CI 91.68%-98.4%), respectively. At this cutoff point around 98.5% of preterm newborn subjects can be correctly identified as preterm by foot length measurement. Also, at this cutoff value, 96.3% of term newborns can be correctly identified as term by foot length measurement. At this foot length value, the positive predictive value was 93%, while the negative predictive value was 99.2%. The false positive rate was 3.7% (3.7% of actual term newborns are classified as preterm by foot length measurement of newborns).

## Discussion

In this study we have shown that, in the resource-poor setting lacking ultrasound and trained manpower, measurement of newborn foot length should be used as a screening tool to identify premature newborns and allow proper early intervention to enhance their survival [21, 22]. Foot length had high sensitivity and specificity in identifying preterm babies, making it a reliable tool in a rural setting [23]. In the present study, it was found that foot length had a statistically significant strong classification power to categorize preterm and term newborns. The diagnostic performance of foot length measurement was very strong with AUC of 0.990 (95% CI 0.978–1.0). Our finding is higher than other studies conducted in Vietnam, Nepal, Surakarta, and Uganda with AUC score based on ROC curve 0.88, 0.683, 0.868 and 0.95, respectively [20, 21, 24, 25]. This is likely attributable to the larger sample size of preterm newborns in the current study.

In the present study, the mean foot length of newborn subjects was found to be 7.41 cm (95% CI 7.32–7.50 cm) which is similar to studies conducted in Vietnam (7.4), Eastern India (7.33) and Aurangabad-India (7.42) [24, 26, 27]; but lower than reported from Uganda, Nepal, and Bengaluru [20, 21, 23]. The difference in mean foot length could be due to genetic, racial or regional (geographic) factors. It could also be due to a difference in measuring instruments (stiff transparent plastic ruler) used in those studies.

There was no statistically significant difference (p = 0.580) in mean foot length measurement between male and female newborn subjects. This finding is supported by the studies conducted in Surakarta and South Africa [25, 28].

The present study has shown that the optimal cutoff point of foot length was found to be 7.35 cm with a sensitivity of 98.5% (95% CI 92.1%-99.7%) and specificity of 96.3% (95% CI 91.6%-98.4%). However, foot length cut-point values vary by setting which were 7.1–8.0 cm in Asia [21, 24–26, 29] and 7.6–8 cm in Africa [20, 22, 28], respectively. A study conducted in Vietnam found that a foot length ≤7.3 cm taken at birth was 80% sensitive and 81% specific in identifying premature (<37 weeks) newborns which is comparable with the cutoff point found in the present study [24]. This finding is also comparable with the study done in Bengaluru reporting the cut-off point for FL of ≤ 7.4 cm with 98.81% sensitivity and 79.09% specificity for identifying preterm babies [22]. Studies conducted in Nepal and India revealed that the operational cut-off for determining preterm newborns was 7.8 cm and 7.75 cm, respectively [21, 26]. This is higher than the cutoff point found in the present study (7.35 cm). A study performed in Surakarta showed that the optimal cutoff foot length for full-term categorization was 7.1 cm with a sensitivity of 75% and specificity of 98.1% which is lower than the cut-off point in the present study [25]. Recent African studies conducted in southern Tanzania and Uganda have used foot lengths of ≤8 cm and ≤7.6 cm with sensitivity and specificity of 93% (95%CI 82%-99%) and 58% (95%CI 53%-62%), and 96% (95%CI 82–100) and 76% (95% CI 73–79), respectively, to identify preterm newborns. These cutoff values are much higher than the present study [20, 22]. The cut-off point of foot length in Indore (7.37 cm) is consistent with the present finding [29]. The difference in operational cutoff foot length value could be due to genetic, racial, regional (geographic) and measuring instrument variation.

**Fig 3:**
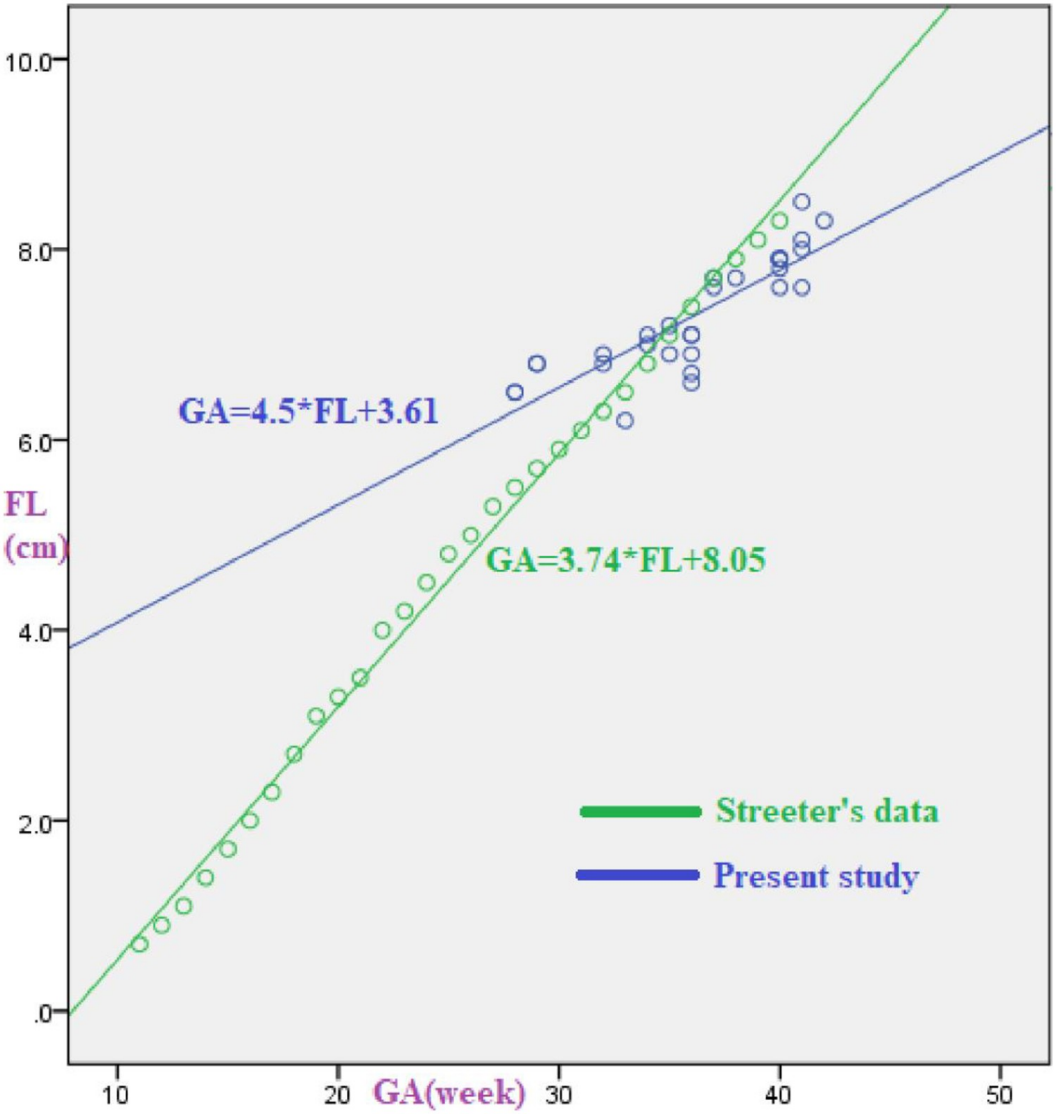
Comparison of the regression line of gestational age(GA) versus foot length (FL) in the present study and Streeter’s data.

In the present study, a statistically significant strong positive correlation was found between gestational age and foot length (r = 0.865 (95% CI 0.832–0.895) and p-value = 0.000). This finding is strongly supported by other studies conducted in Eastern India, Bengaluru, South Africa, and India, where foot length and gestational age had a strong positive correlation [23, 26, 28, 30]. However, a report in Surakarta showed a weaker statistically significant correlation (r = 0.533; P = 0.000) [25]. Accordingly, studies in Belgaum, Indore, north India, and Nagpur observed a significant strong positive correlation between FL and GA with r -value 0.988, 0.99, 0.975, and 0.960 where the coefficient of correlation is greater than the current study [29, 31–33]. In the present study, GA can be estimated using the regression equation: GA = 4.5*FL + 3.61. However, in Streeter’s data [34] the regression equation was: GA = 3.74*FL + 8.05 and in the Karnataka, Indian study [30] it was: GA = 3.8*FL + 7.68. One cm increase in the FL of newborns increases the gestational age by 4.5 weeks in our study, 3.79 weeks in Karnataka, Indian study and 3.74 weeks in Streeter’s study. A linear association was obtained when FL was plotted against GA, which is comparatively similar to the linear curve obtained in the Streeter’s. An intercept between the 2 regression lines was also close to the threshold FL of 7.35 (Fig 3).

Foot length cannot be used for those of SGA or multiple gestation. As this was a hospital based study, there is also a need to validate the tool in the community setting (with community health workers) for which its use is intended. Additionaly, the study did not validate against other means of determining gestational age like use of ultrasonography which may have different results.

In conclusion, the foot length measurement taken within 24 hours of birth could be used to estimate GA. It had a high sensitivity and specificity in identifying preterm newborns making it a reliable tool that could be used in a rural setting. There was no statistically significant difference in mean foot length measurement between male and female newborn subjects.

## Supporting information

S1 File Informed verbal consent. (DOCX)
