# Supplementary material for: Measuring newborn foot length to estimate gestational age in a high risk Northwest Ethiopian population
Source: PLoS One. 2020 Aug 27;15(8):e0238169. doi: 10.1371/journal.pone.0238169 (PMC7451509; doi:10.1371/journal.pone.0238169)
Supplement: S1 File — (DOCX) [file pone.0238169.s001.docx]

Informed verbal consent

Dear Participants

My name is -------------------------------- I am a data collector for Mr. Nega Dagnew thesis
work for partial fulfillment of master degree graduation in Human Anatomy on the topic of measuring newborn foot length to identify preterm neonates at the University of Gondar Comprehensive Specialized Hospital, Northwest Ethiopia. This research will be important to assess the use of newborn foot length as a screening tool to identify preterm newborns and associated factors. So, your newborns foot length measure will be greatly important to assess the use of newborn foot length as a screening tool to identify preterm newborns and factors associated with it. I assure you that all your newborns data will be completely confidential and none your newborns data will be reported separately to anybody. It is your full right to participate or refuse in the study. There are no risks to your newborn associated with participation in this study.

We would greatly appreciate your help in participating to this study.

Are you willing to participate in this study?
Yes No
Code No. ----------------
Thank you for your genuine cooperation!!
